# Comment on: A cross-sectional analysis of liver disease–hypertension comorbid mortality trends in the United States, 1999–2023

**DOI:** 10.1097/MS9.0000000000005558

**Published:** 2026-08-21

**Authors:** FNU Mayori, Arthi Roy

**Affiliations:** a Department of Medicine, Peoples University of Medical and Health Sciences for Women, Pakistan; b Department of Medicine, Pabna Medical College, Pabna, Bangladesh

*Dear Editor*,

I read with great interest the article by Mazhar *et al*, “A cross-sectional analysis of liver disease–hypertension comorbid mortality trends in the United States, 1999–2023”^[^1^]^. The article provides valuable insights into temporal trends in deaths co-listed with liver disease and hypertension using a large national mortality database. The authors are to be congratulated for this comprehensive assessment of demographic and geographic patterns over a 25-year period. However, several methodological considerations may further improve the interpretation of these results.

First, the broad ICD-10 categories allowed complete case ascertainment. However, combining all liver diseases (K70–K76) into one group for analysis may mask important liver-disease subtype-specific mortality patterns. There is evidence that different liver disease sub-types follow very different trajectories over time. For example, Ryu *et al* showed increasing mortality trends for alcoholic fatty liver disease, alcoholic hepatitis, and alcoholic cirrhosis but decreasing trends for alcoholic hepatic failure and unspecified alcoholic liver disease^[^2^]^. Such heterogeneity suggests that aggregation of diverse liver disease entities may mask the specific conditions driving observed mortality increases and limit the clinical interpretability of population-level findings.

Moreover, age-adjusted mortality rates are a good way to adjust for demographic changes over time, but age-stratified analyses were not reported. Therefore, it is not clear whether this increase in co-listed liver disease–hypertension mortality was primarily in younger, middle-aged, or older adults. Prior population-based studies have shown that patterns of mortality from chronic liver disease vary considerably by age-group and etiology, highlighting the necessity of age-specific analyses to identify populations most responsible for mortality trends^[^3^]^.

Lastly, one factor is the changing clinical recognition of metabolic dysfunction-associated steatotic liver disease (MASLD). Recent international consensus efforts have highlighted the enhanced identification and classification of MASLD, raising awareness of metabolic dysfunction as a key driver of liver disease^[^4^]^. Therefore, improved diagnostic detection may have increased the likelihood of liver disease being recorded on death certificates. This increased detection may have contributed to the observed increase in liver disease–hypertension mortality co-listing, independent of the underlying disease burden.

Overall, this study adds important population-based surveillance data on the patterns of co-listing of liver disease and hypertension. Additional analyses with disease-specific subtypes, age-stratified trends, and the potential impact of evolving MASLD recognition may help to better understand the factors underlying the observed increase in mortality.

## Data Availability

Data sharing is not applicable to this article, as no new data were generated or analyzed.
